# Supplementary material for: MSH2 shapes the meiotic crossover landscape in relation to interhomolog polymorphism in Arabidopsis
Source: EMBO J. 2020 Sep 16;39(21):e104858. doi: 10.15252/embj.2020104858 (PMC7604573; doi:10.15252/embj.2020104858)
Supplement: Supplementary file 1 — Appendix [file EMBJ-39-e104858-s001.pdf]

MSH2 shapes the meiotic crossover landscape in relation to interhomolog polymorphism in Arabidopsis

Alexander R. Blackwell, Julia Dluzewska, Maja Szymanska-Lejman, Stuart Desjardins, Andrew J. Tock, Nadia Kbiri, Christophe Lambing, Emma J. Lawrence, Tomasz Bieluszewski, Beth Rowan, James D. Higgins, Piotr A. Ziolkowski and Ian R. Henderson

Appendix:

Appendix Figures S1- S12

Appendix Tables S1 - S13

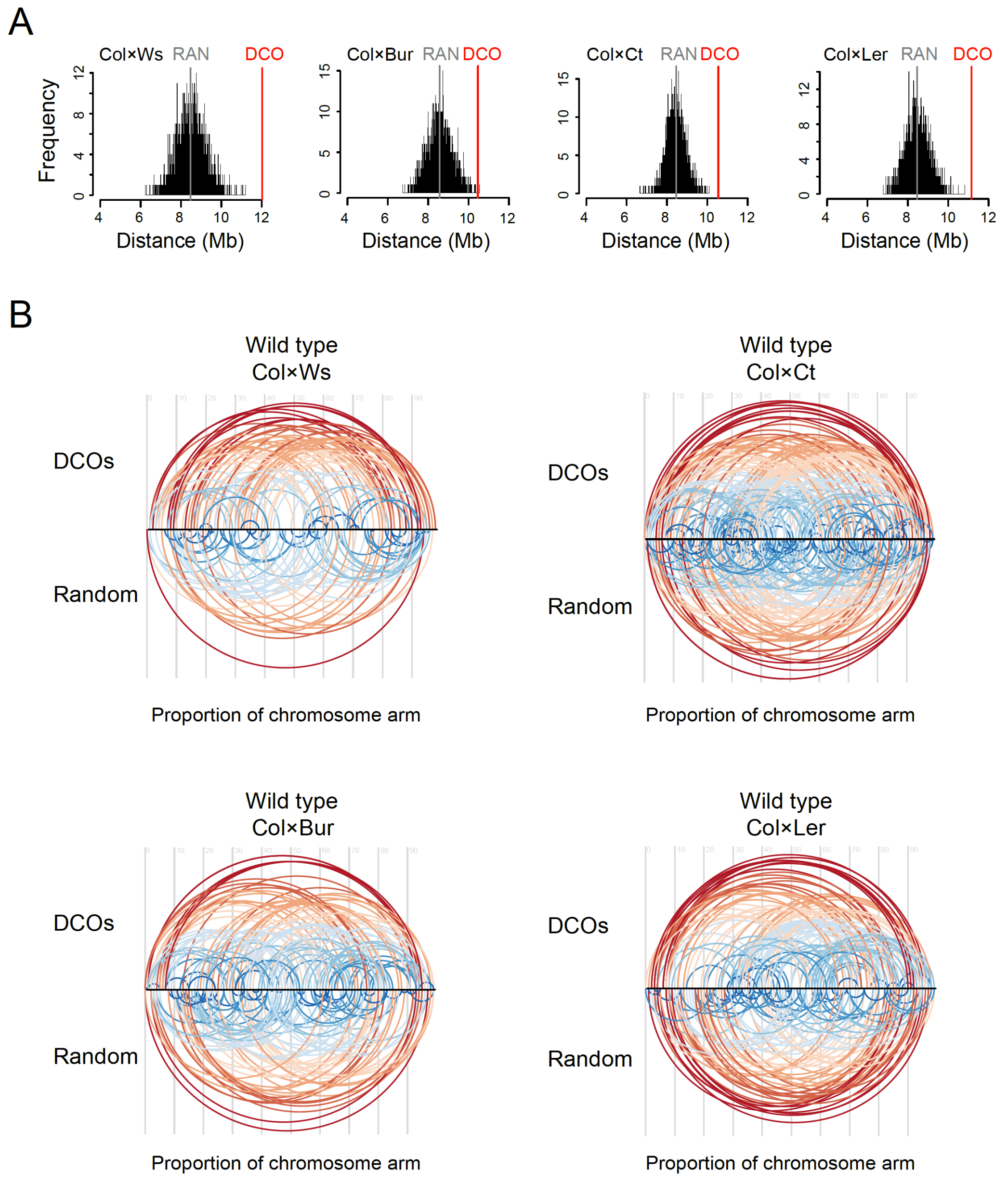

Appendix Figure S1. Analysis of double crossover spacing in Col×Ler, Col×Bur, Col×Ws and Col×Ct F_2 populations. A. Histograms showing the mean distance (megabases, Mb) of observed double crossovers (DCOs, red), compared to 2,000 matched sets of randomly generated distances (RAN, black). The means of the random sets are shown in grey. *Cis*-DCOs were identified from F_2 genotyping data, by filtering for parental-heterozygous-parental genotype transitions (e.g. Col-Het-Col or Ler-Het-Ler in Col×Ler F_2 individuals) (Drouaud *et al*, 2005; Rowan *et al*, 2019; Lambing *et al*, 2020a). **B.** Diagrams showing spacing of identified *cis* DCOs along the proportional physical length of chromosomes (%). DCOs are connected via arcs and color-coded proportional to the distance between them (red=greatest, blue=smallest).

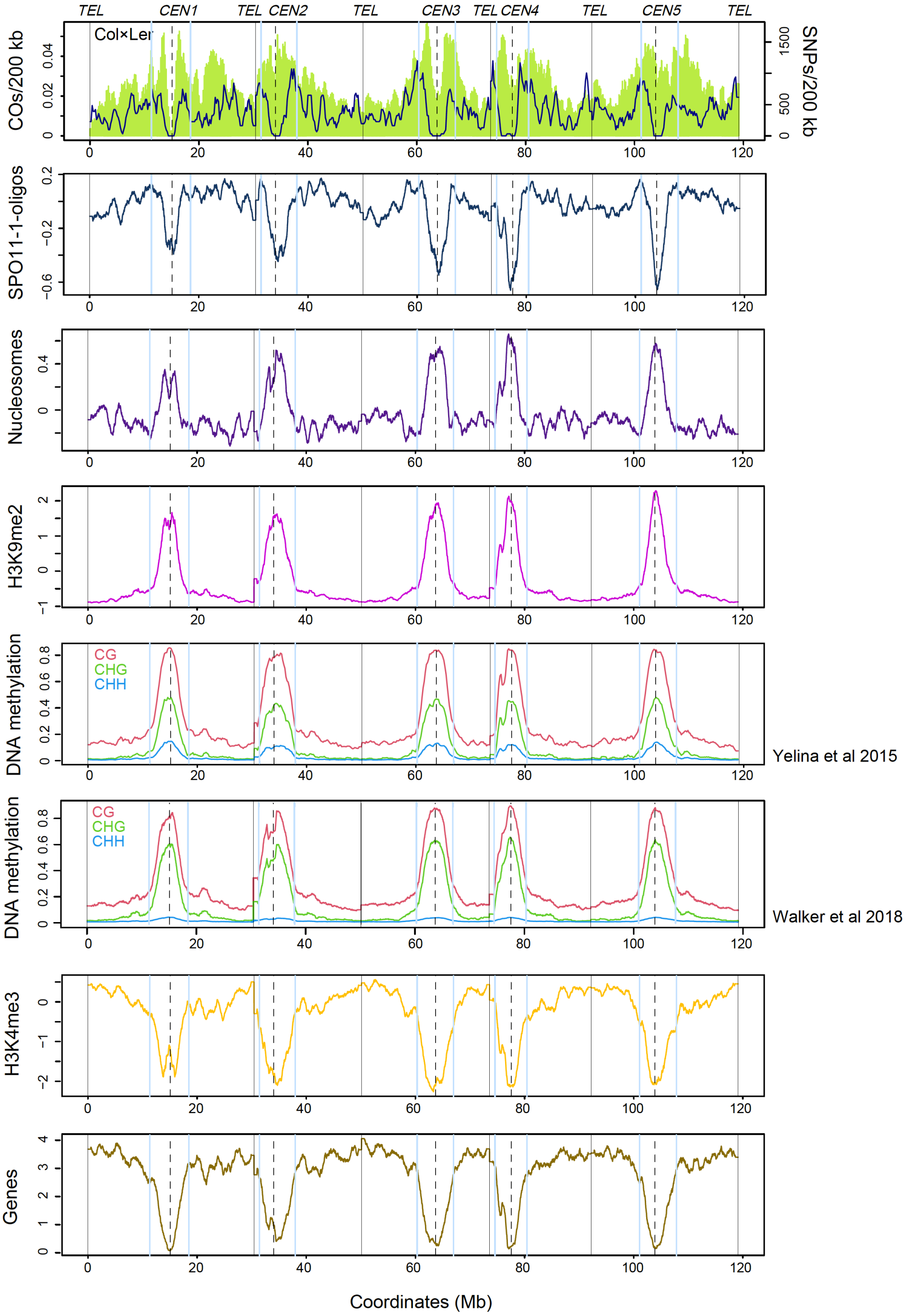

Appendix Figure S2. Meiotic recombination, polymorphism and chromatin landscapes throughout the Arabidopsis genome. Crossovers per 200 kb normalized by F_2 individuals is plotted along the Arabidopsis chromosomes derived from a Col×Ler cross. Col/Ler SNPs per 200 kb are shaded in green. Note that the upper panel showing crossover frequency and SNPs is reproduced from Fig. 1B for ease of comparison. Beneath are plots of SPO11-1-oligos, nucleosomes (MNase-seq), H3K9me2, DNA methylation (CG, CHG and CHH contexts), H3K4me3 and gene density (Choi *et al*, 2018; Lambing *et al*, 2020b; Yelina *et al*, 2015; Choi *et al*, 2016). DNA methylation is shown from somatic and meiotic cells (Walker *et al*, 2018; Yelina *et al*, 2015). The positions of telomeres (*TEL*) and centromeres (*CEN*) are indicated. The boundaries of the pericentromeres are indicated by vertical light blue lines, flanking the centromere assembly gaps (vertical dashed black lines) (Underwood *et al*, 2018).

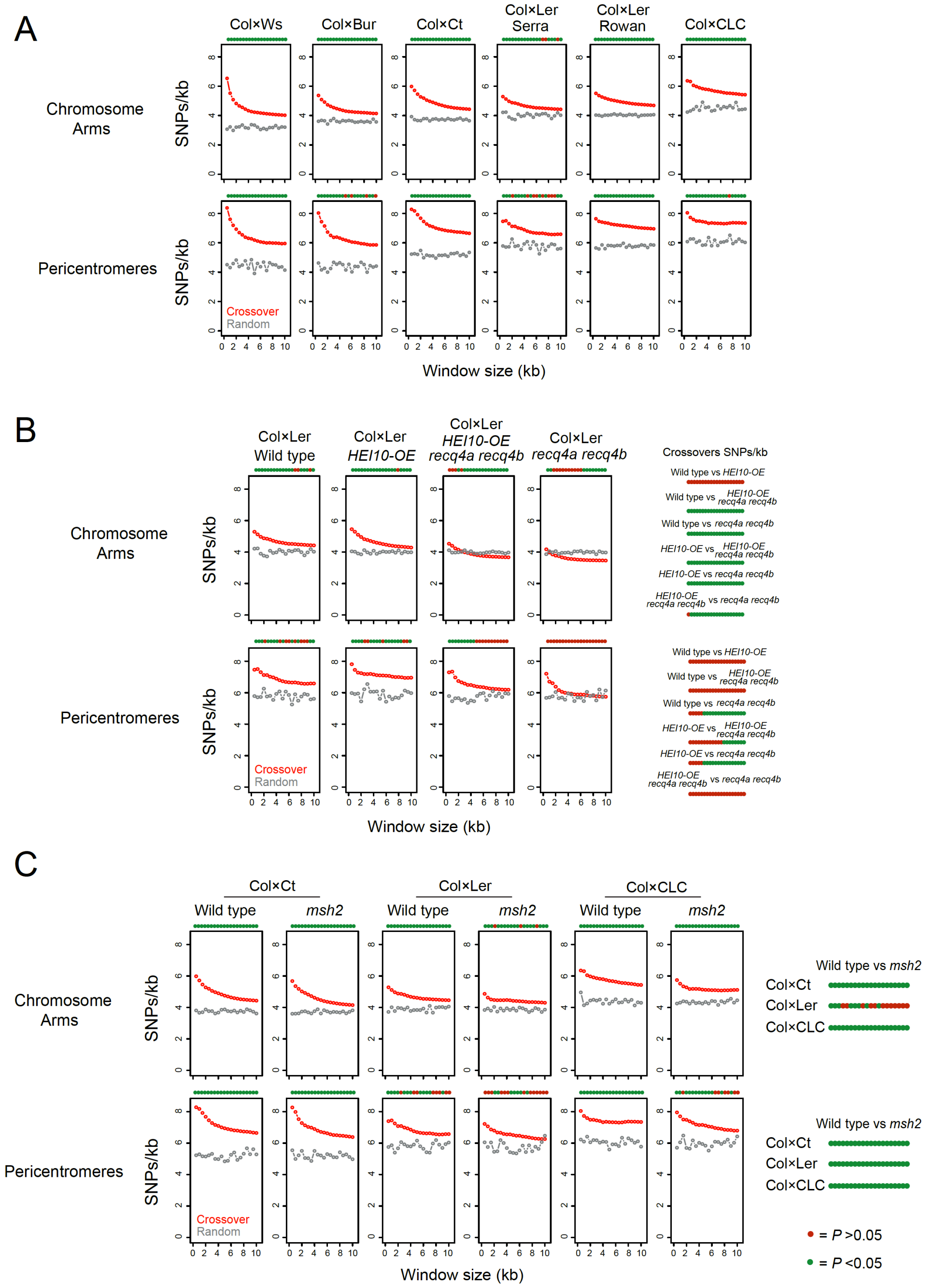

Appendix Figure S3. Crossovers are positively associated with SNP density at the local scale in the chromosome arms and the pericentromeres. A. SNPs/kb in physical windows of increasing size (kb) around crossover midpoints (red), or for matched randomly chosen positions (grey), analysed separately according to location within the chromosome arms or the pericentromeres (Underwood *et al*, 2018). Printed above the plot for each window is a circle colored green if crossover SNPs/kb values are significantly different to random (P<0.05), or red if not (P>0.05) (Bonferroni adjusted t-tests). The population (Col×Ws, Col×Bur (Lawrence *et al*, 2019), Col×Ct, Col×Ler (Serra *et al*, 2018; Rowan *et al*, 2019) and Col×CLC) is printed above each plot. **B.** As for A, but analysing Col/Ler SNPs/kb around crossovers from wild type, *HEI10-OE*, *recq4a recq4b* or *HEI10-OE recq4a recq4b* (Serra *et al*, 2018). All populations were generated from Col×Ler hybrids. To the right are plots showing significance of SNPs/kb differences between crossover sets, across the windows tested. **C.** As for A but analyzing SNPs/kb in wild type and *msh2* in the indicated cross.

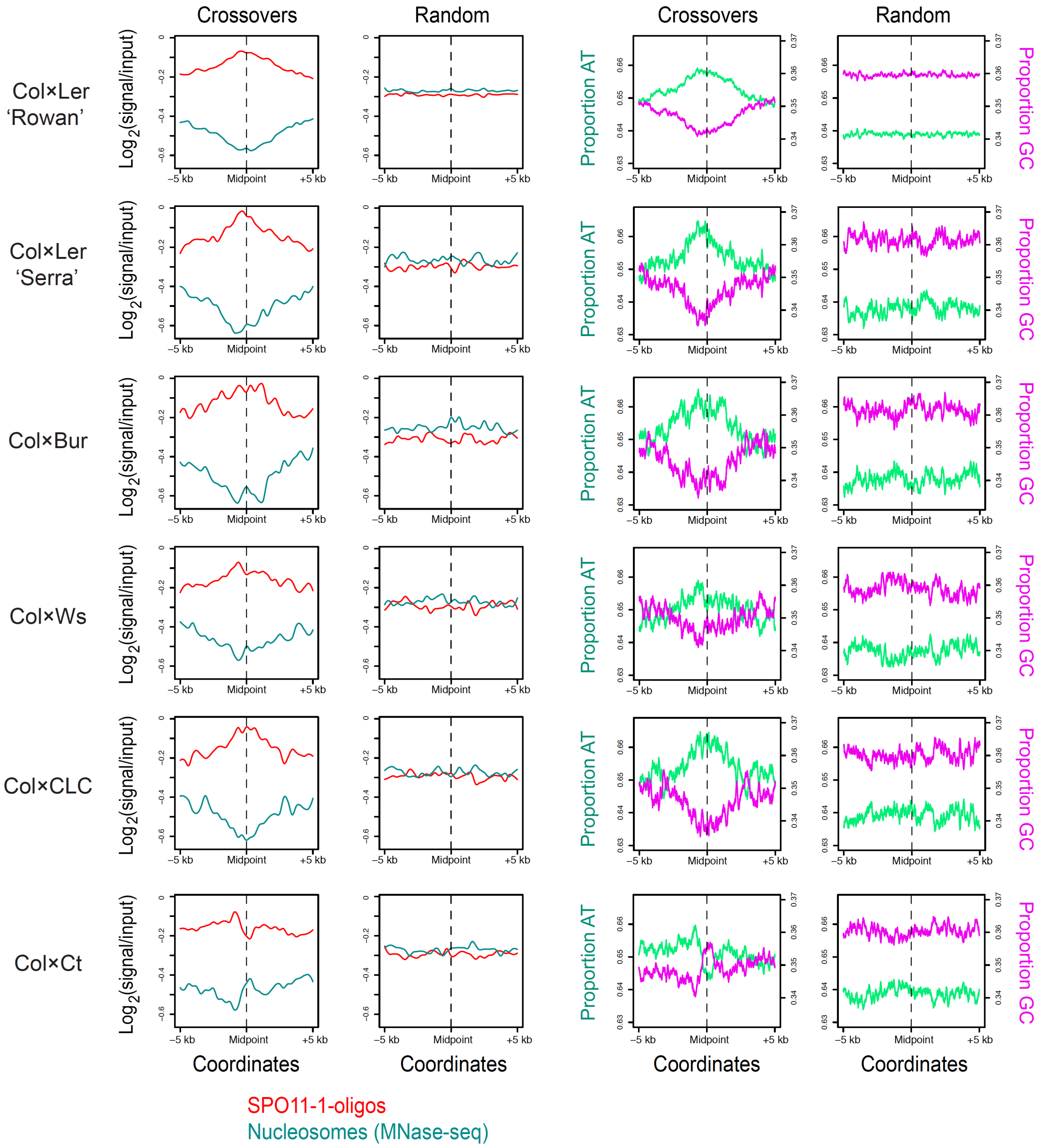

Appendix Figure S4. SPO11-1-oligonucleotides, nucleosome occupancy and base composition around wild type crossovers. 10 kb windows surrounding crossover midpoints, or the same number of randomly selected positions, were analysed for SPO11-1-oligos (log_2(SPO11-1-oligos/gDNA), red) or nucleosome occupancy (log_2(MNase-seq/gDNA), blue) (Choi *et al*, 2018). Base composition was analysed in the same windows and the proportion of AT (green) and GC (purple) plotted.

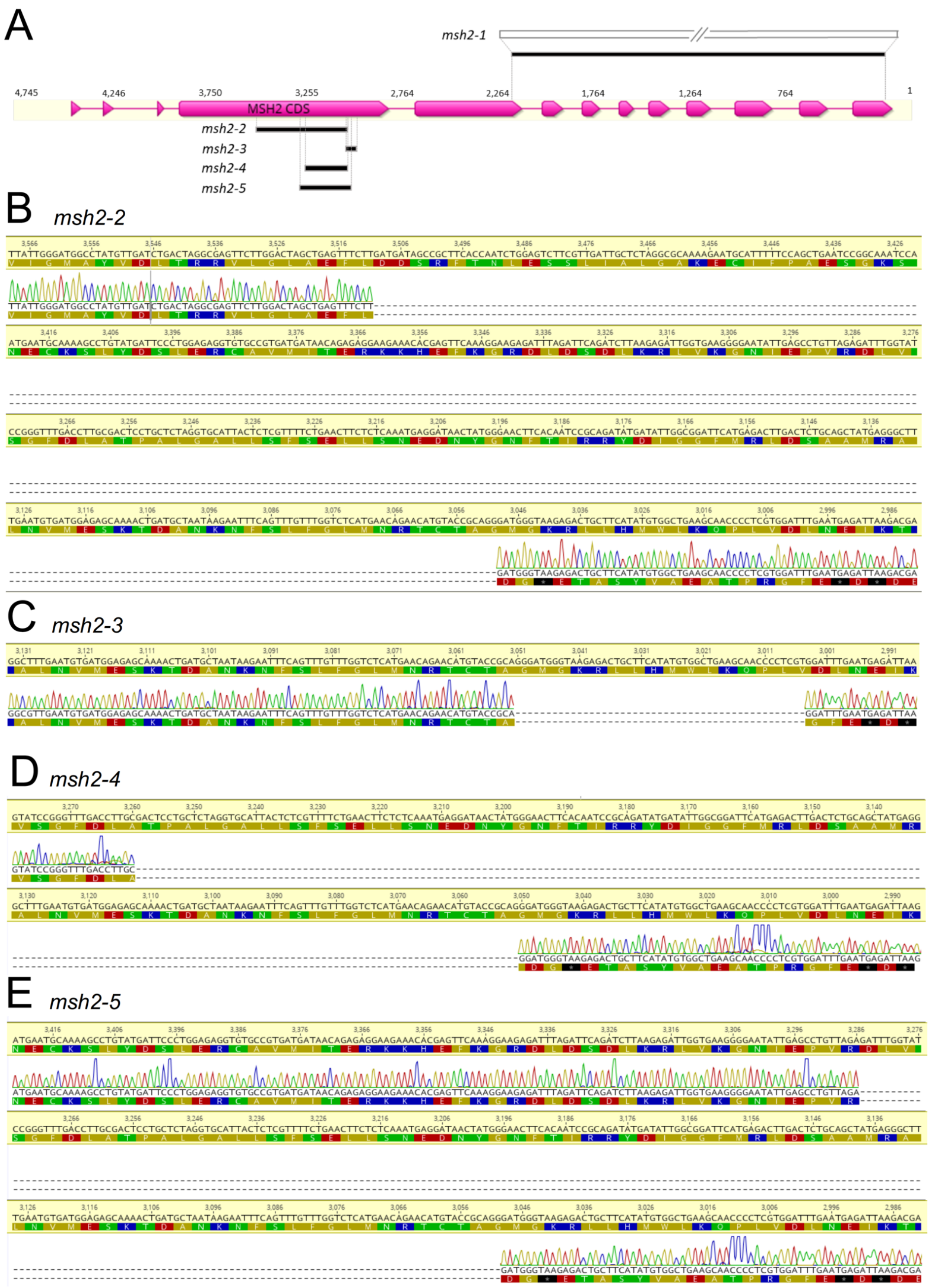

Appendix Figure S5. Characterization of *msh2* mutations generated via CRISPR/Cas9 mutagenesis. A. *MSH2* gene organization showing the position of mutations isolated in this study. Pink bars represent exons, black bars represent deletions in the indicated mutants, and the white bar represents the *msh2-1* T-DNA insertion. The *msh2-1* T-DNA is also associated with a deletion of 2.1 kilobases. **B.** Genomic DNA sequence from the *msh2-2* mutant aligned to wild type *MSH2* genomic sequence. The translation for the reference sequence and the mutant allele is shown, with black representing stop codons. **C.** As for B, but showing *msh2-3*. **D.** As for B, but showing *msh2-4*. **E.** As for B, but showing *msh2-5*.

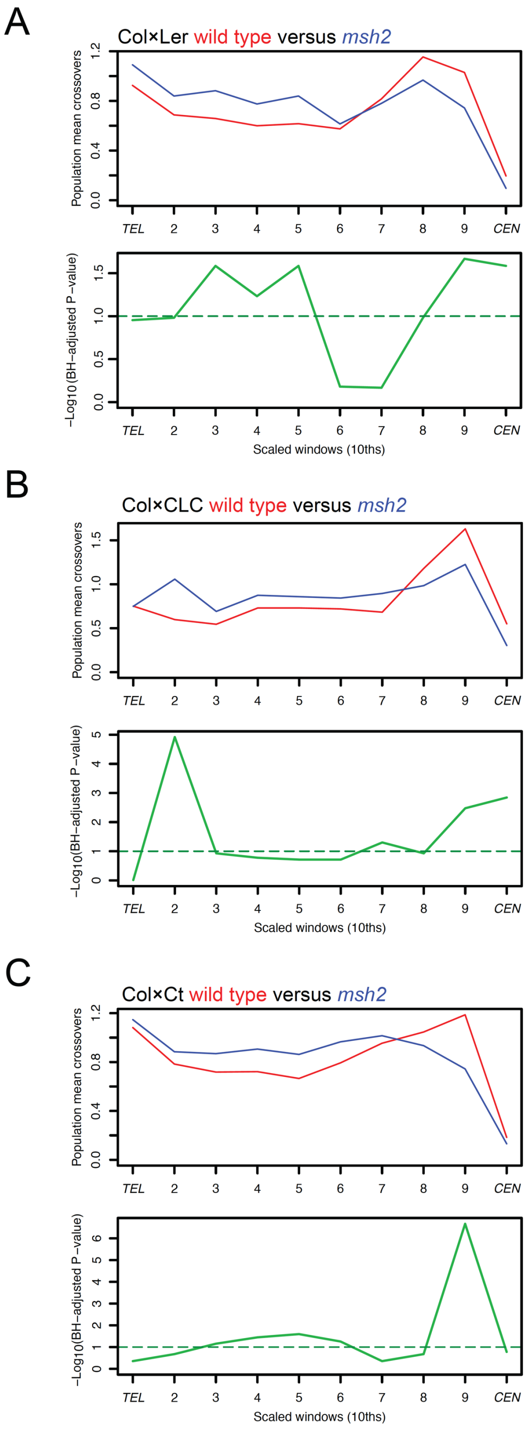

Appendix Figure S6. Significance of crossover frequency changes along the telomere to centromere chromosomes axes in wild type versus *msh2*. A. Crossover frequency in 10 scaled windows along the proportional length of all chromosome arms from telomeres (*TEL*) to centromeres (*CEN*) in Col×Ler wild type (red) and *msh2* (blue) F_2 populations. The Benjamini-Hochberg (BH) multiple-testing-corrected *P*-values (green) are plotted beneath, which are derived using a Poisson model to compare crossover counts in each scaled window, summed across all chromosome arms, between wild type and *msh2* populations. Horizontal dashed lines correspond to a BH multiple-testing-corrected *P*-value threshold of $-\log_{10}(0.1)$ (green). **B.** As for A, but comparing wild type and *msh2* in a Col×CLC background. **C.** As for A, but comparing wild type and *msh2* in a Col×Ct background.

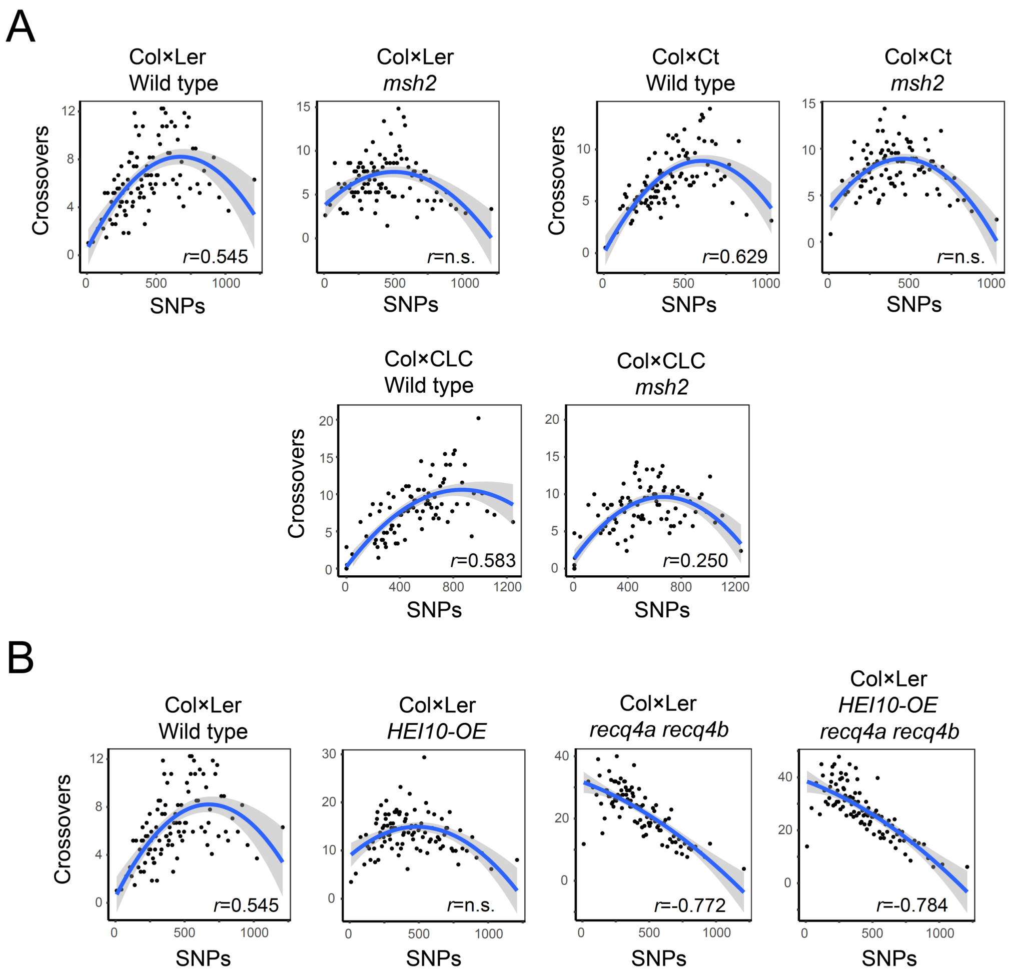

Appendix Figure S7. Correlations between crossover frequency and SNP density in wild type, *msh2, HEI10-OE, recq4a recq4b* and *HEI10-OE recq4a recq4b*. A. Crossover frequency normalised by the number of F_2 individuals, and SNP density were calculated in 100 kilobase (kb) adjacent windows for each wild type and *msh2* population. Centromeric regions were excluded from analysis (Underwood *et al*, 2018). Windows were ranked into SNP density percentiles and values plotted. Spearman's rank correlation coefficient (*r*) between SNP density and crossover frequency is printed inset. Trend lines were fitted in ggplot using generalized additive model (GAM) with the formula y~poly(x,2). Note that the wild type Col×Ler, Col×Ct and Col×CLC datasets are reproduced from Figure 2A. **B.** As for A but analyzing crossovers in Col/Ler wild type, *HEI10-OE*, *recq4a recq4* and *HEI10-OE recq4a recq4b.* Note that the wild type Col×Ler dataset is reproduced from Figure 2A.

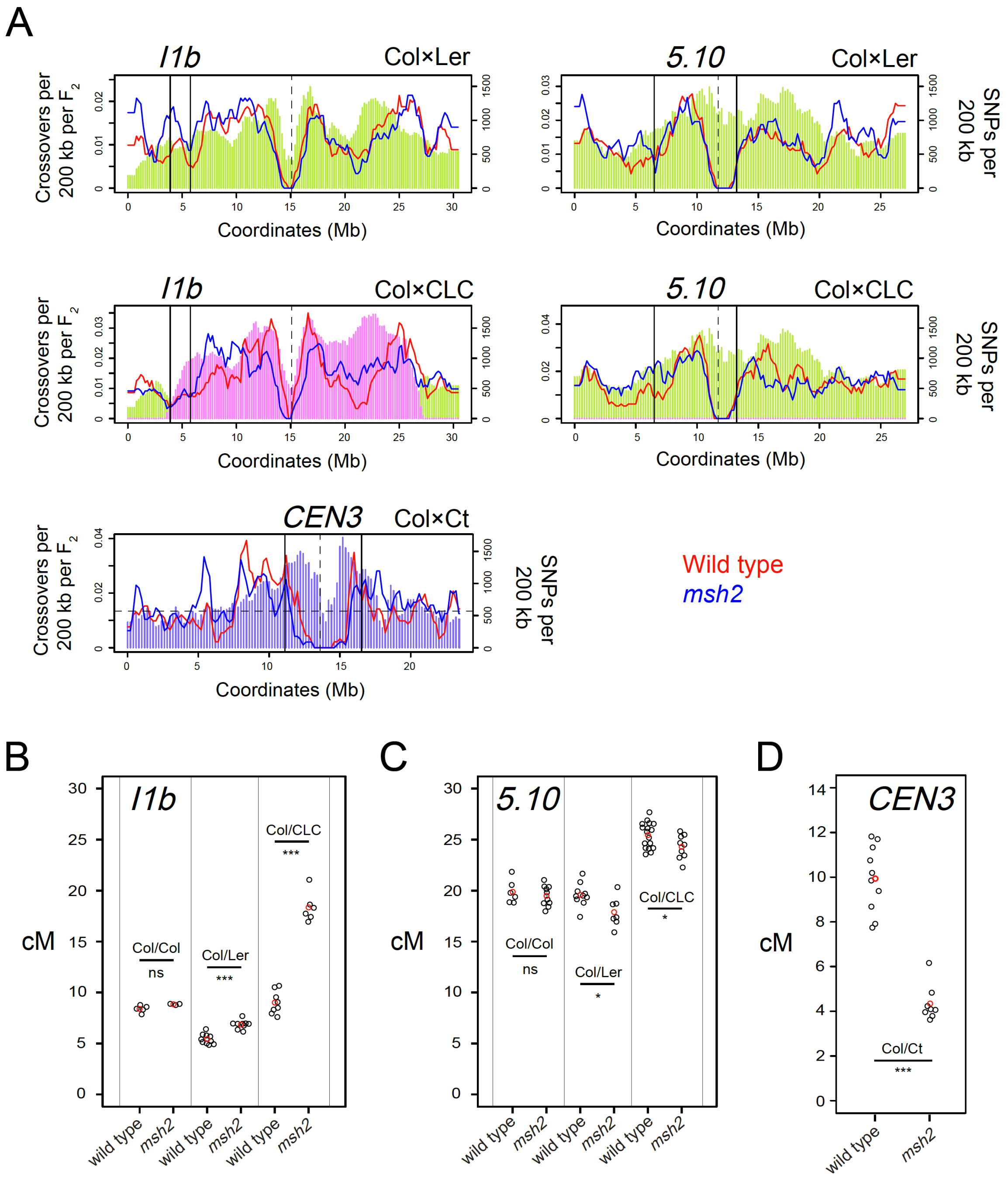

Appendix Figure S8. Measuring crossover frequency using FTLs in wild type and *msh2* in inbred, Col×Ler, Col×CLC and Col×Ct backgrounds. A. Crossovers per 200 kb per F_2 plotted along Arabidopsis chromosomes 1, 3 and 5 from wild type (red) and *msh2* (blue), from Col×Ler, Col×CLC and Col×Ct hybrids. SNP density per 200 kb is shaded. The positions of the *I1b*, *5.10* and *CEN3* FTL T-DNAs are indicated by black vertical lines. The centromere positions are indicated by vertical dashed lines. **B.** *I1b* genetic distance (cM) measured in Col×Col inbred or Col×Ler and Col×CLC hybrids, in wild type and *msh2*. Black dots represent replicates and red dots represent genotype means. Statistical significance was assessed using X^2 tests and *P* values are indicated as <0.05 (*), 0.01 (**) and 0.001 (***), or not significant (ns). **C.** As for C, but for FTL interval *5.10*. **D.** As for A, but for FTL interval *CEN3* in Col×Ct hybrids.

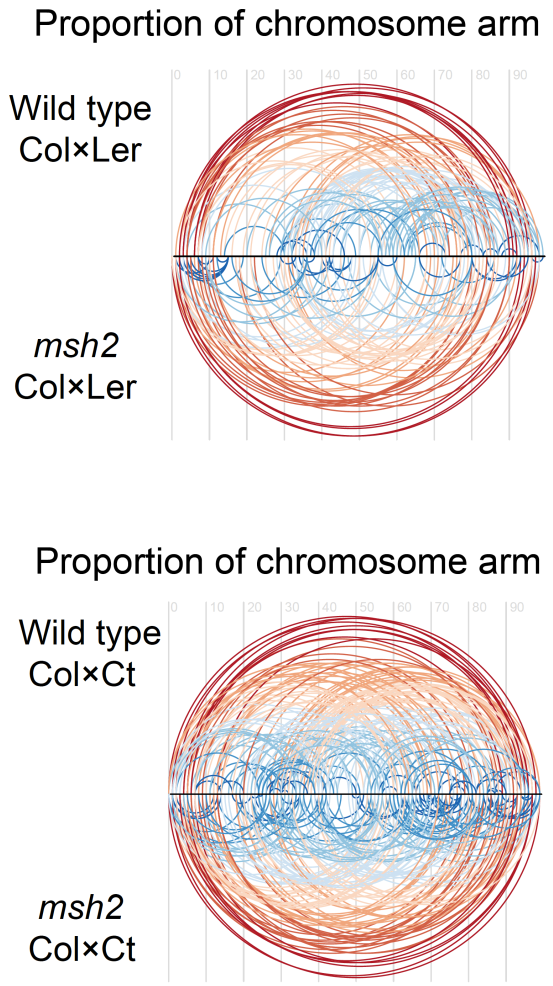

Appendix Figure S9. Double crossover analysis in wild type and *msh2*. Diagrams showing spacing of identified *cis* DCOs along the proportional physical length of chromosomes (%). C*is*-DCOs were identified from F_2 genotyping data, by filtering for parental-heterozygous-parental genotype transitions (e.g. Col-Het-Col or Ler-Het-Ler in Col×Ler F_2 individuals) (Drouaud *et al*, 2005; Rowan *et al*, 2019; Lambing *et al*, 2020a). DCOs are connected via arcs and proportionally color-coded according to the distance between them (red=greatest, blue=smallest).

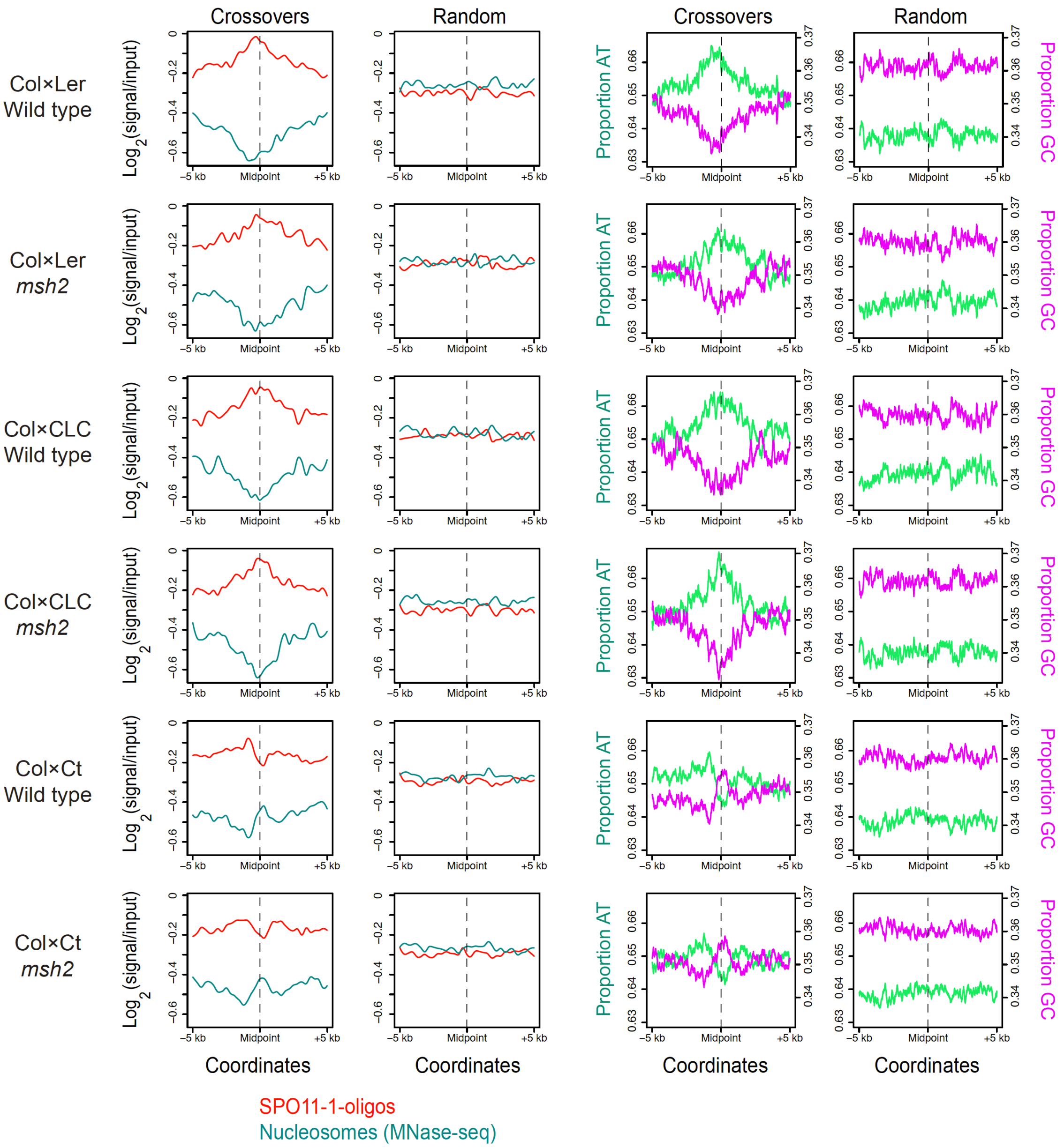

Appendix Figure S10. SPO11-1-oligonucleotides, nucleosome occupancy and base composition around wild type and *msh2* crossovers. 10 kb windows surrounding crossover midpoints, or the same number of randomly selected positions, were analysed for SPO11-1-oligos ($\log_2$(SPO11-1-oligos/gDNA), red) or nucleosome occupancy ($\log_2$(MNase-seq/gDNA), blue) (Choi *et al*, 2018). Base composition was analysed in the same windows and the proportion of AT (green) and GC (purple) plotted. Note that the wild type Col×Ler, Col×Ct and Col×CLC datasets are reproduced from Appendix Figure 4.

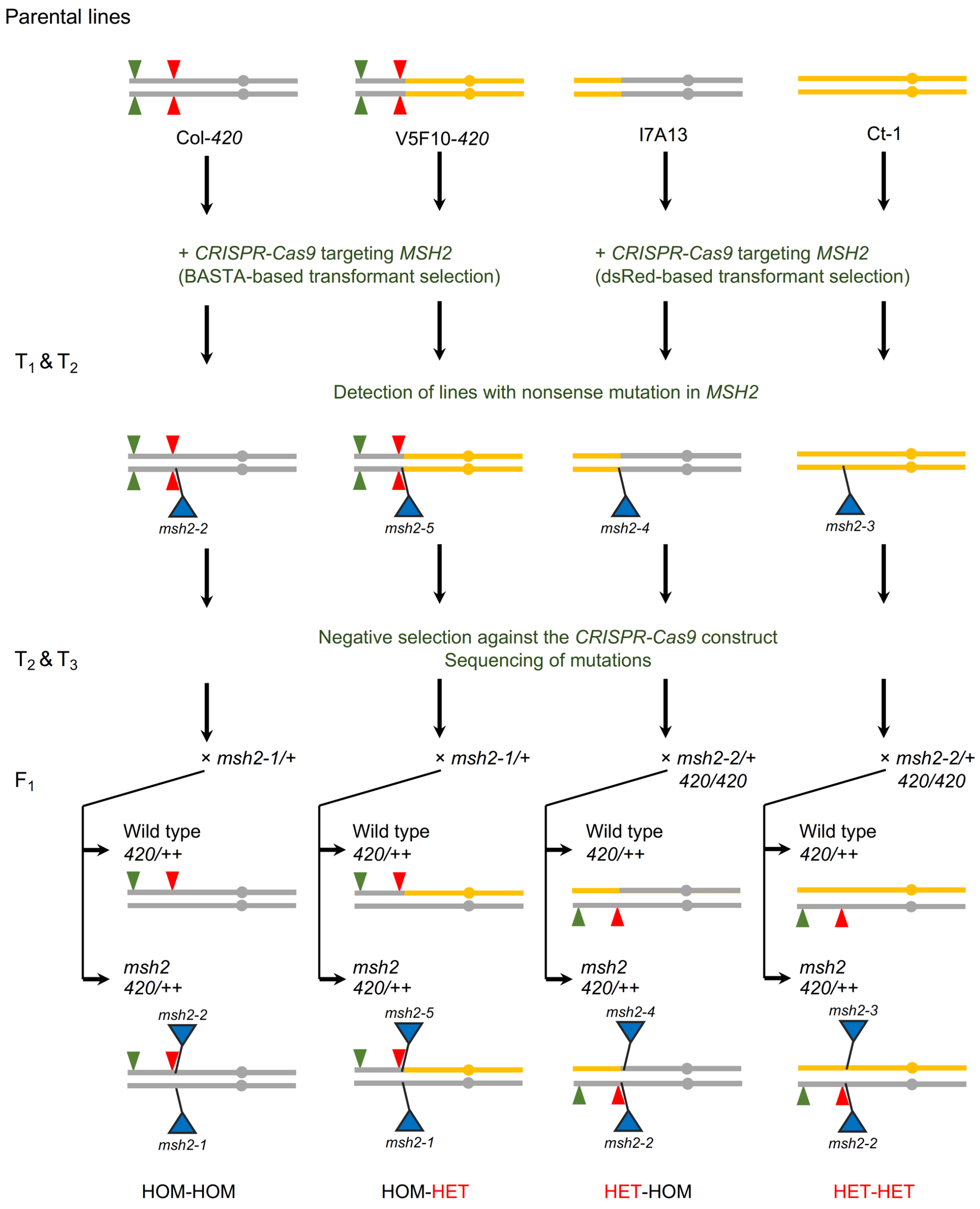

Appendix Figure S11. Generation of *msh2* mutants with varying patterns of Col/Ct heterozygosity and carrying the *420* FTL. Parental lines with fixed regions of Col and Ct homozygosity along chromosome 3 were previously characterized (Ziolkowski *et al*, 2015). Chromosome ideograms represent sequence polymorphism along chromosome three, with grey corresponding to Col and yellow corresponding to Ct. The locations of the *420* FTLs (red and green triangles) used to measure crossover frequency are indicated. Note that the *MSH2* gene is closely linked to *420*.

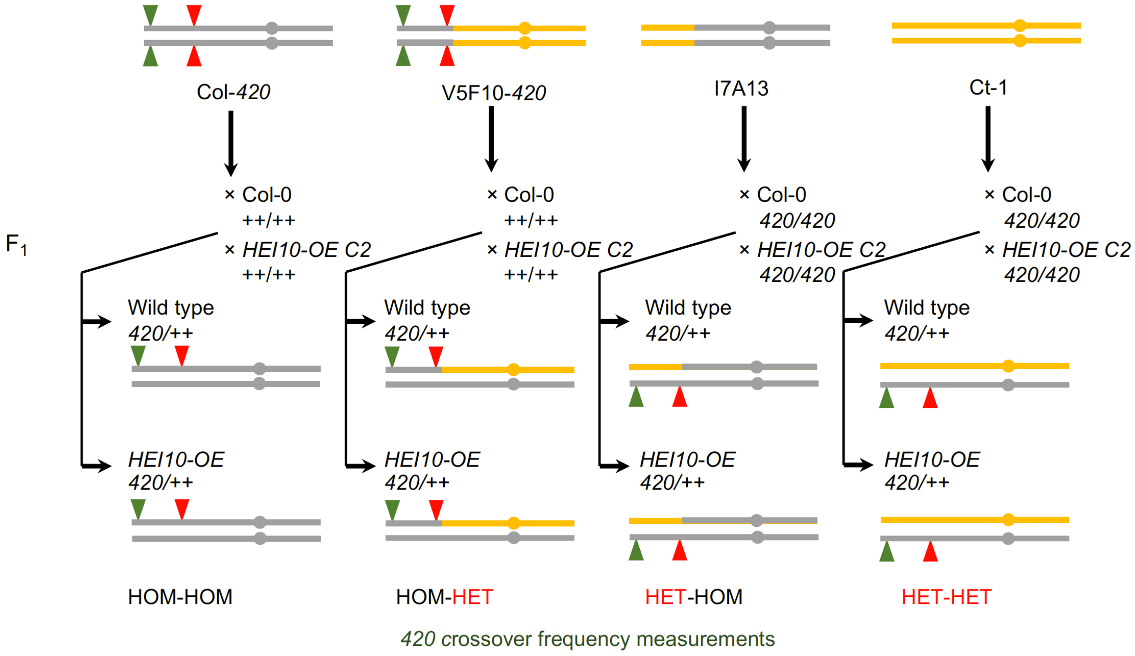

Appendix Figure S12. Generation of *HEI10-OE* overexpression lines with varying patterns of Col/Ct heterozygosity and carrying the FTL *420*. Parental lines with fixed regions of Col and Ct homozygosity along chromosome three were previously characterized (Ziolkowski *et al*, 2015). These lines were crossed with the *HEI10-OE* C2 line (Ziolkowski *et al*, 2017). The locations of the *420* FTLs (red and green triangles) used to measure *420* crossover rate are indicated.

Appendix Table S1. Single nucleotide polymorphism (SNP) numbers between the accessions used for crossover mapping. SNPs were identified for the Ler, Bur, Ws, Ct and CLC backgrounds relative to the Col TAIR10 reference. Identified SNP numbers are shown for each chromosome and in total for the indicated comparisons. SNPs/kb values are assuming a genome size of 125,000 kb.

Chromosome	Col×Ws	Col×Bur	Col×Ct	Col×Ler	Col×CLC
Chr 1	97,894	111,749	117,181	125,326	156,778
Chr 2	72,785	73,221	71,313	86,603	78,608
Chr 3	85,465	86,621	91,108	99,167	127,386
Chr 4	54,965	69,992	81,292	84,733	75,646
Chr 5	102,721	104,778	114,793	124,005	124,005
Total	413,830	446,361	475,687	519,834	562,423
SNPs/kb	3.31	3.57	3.81	4.16	4.50

Appendix Table S2. Percentage of single nucleotide polymorphisms shared between accessions used for crossover mapping. SNPs were identified for the Ws, Bur, Ct, Ler and CLC backgrounds relative to the Col TAIR10 reference. The 'Total' column lists the total SNPs identified for the accessions listed in the first Column. The remaining columns show the proportion of SNPs shared with the indicated genotypes, relative to Col.

Genotype	Total	Ws	Bur	Ct	Ler	CLC
Ws	413830	-	0.42	0.44	0.47	0.41
Bur	446361	0.38	-	0.41	0.43	0.39
Ct	475687	0.38	0.39	-	0.46	0.40
Ler	519834	0.38	0.37	0.42	-	0.42
CLC	562423	0.30	0.31	0.33	0.55	-

Appendix Table S3. Crossovers mapped by genotyping-by-sequencing of wild type and *msh2* F_2 populations. The total number of crossovers analysed in each population are listed in total and per chromosome. The number of F_2 individuals analysed and the average number of crossovers per F_2 are listed. The average width (bp) for crossovers identified in each population is listed. For the Col×Ler maps two data sets using 17,077 crossovers ('Rowan' (Rowan *et al*, 2019)) and 1,809 crossovers ('Serra' (Serra *et al*, 2018)) were analyzed. The Col×Bur data was reported as a control for *taf4b* (Lawrence *et al*, 2019). The Col×Ler and Col×CLC data used as wild type controls for *msh2* have a lower crossover number due to additional masking of Col introgressions required for comparison with *msh2*.

Genotype	Total	Chr1	Chr2	Chr3	Chr4	Chr5	F_2	Width (bp)	CO/F_2
Col×Ler Rowan	17,077	4,153	3,021	3,191	2,825	3,887	2,182	3,261	7.83
Col×Ler Serra	1,840	437	326	345	309	423	245	1,045	7.51
Col×Bur	1,396	341	223	259	251	322	180	653	7.76
Col×Ws	1,485	379	265	277	232	332	188	2,262	7.90
Col×CLC	1,587	420	233	279	264	391	189	725	8.40
Col×Ct	2,478	617	438	485	394	544	305	1,161	8.12
**Genotype**	**Total**	**Chr1**	**Chr2**	**Chr3**	**Chr4**	**Chr5**	**F_2**	**Width (bp)**	**CO/F_2**
Col×Ler WT	1,739	434	320	276	295	414	240	1,036	7.25
Col×Ler *msh2*	1,426	362	270	209	228	357	187	1,260	7.63
Col×CLC WT	1,534	420	233	226	264	391	189	727	8.12
Col×CLC *msh2*	1,620	421	253	278	261	407	191	1,056	8.48
Col×Ct WT	2,478	617	438	485	394	544	305	1,161	8.12
Col×Ct *msh2*	2,702	675	456	540	425	606	320	1,526	8.44

Appendix Table S4. Correlation between crossover frequency with varying physical window sizes in wild type F_2 populations. Varying window sizes (kb) were used to calculate crossover frequency in the indicated populations throughout the genome. These values were used to calculate Spearman's rank correlation coefficient.

		Windows (kb)					
**Crossovers**	**Crossovers**	**10**	**50**	**100**	**200**	**500**	**1000**
Col×Ler (Serra)	Col×Ler (Rowan)	0.544	0.776	0.856	0.904	0.932	0.935
Col×Ler	Col×Bur	0.233	0.496	0.615	0.712	0.740	0.703
Col×Ler	Col×Ws	0.230	0.428	0.509	0.580	0.646	0.681
Col×Ler	Col×CLC	0.268	0.460	0.539	0.602	0.660	0.636
Col×Ler	Col×Ct	0.267	0.562	0.698	0.796	0.864	0.881

Appendix Table S5. Generalized linear model showing the relationship between crossovers, SPO11-1-oligos and chromatin. The incidence of one or more crossover within 534,775 Col/Ler SNP intervals was treated as a categorical variable (Serra *et al*, 2018). SPO11-1-oligo, nucleosome occupancy (MNase-seq), H3K4me3, DNA methylation and SNPs/kb were calculated in the same intervals as explanatory variables. For SNP density we calculated a rolling average of SNPs/kb with a 1 base pair step and used these values to calculate mean SNPs per interval. The width of the intervals was also considered in the model, with the formula:

Crossovers ~ (SPO11-1-oligos + nucleosomes + H3K4me3 + DNA methylation + SNPs/kb + width)^^{2}

The final stepAIC-selected model was:

Crossovers ~ SPO11-1 + nucleosomes + H3K4me3 + DNA methylation + SNPs/kb + width + SPO11-1:nucleosomes + SPO11-1:DNA methylation + SPO11-1:width + nucleosomes:H3K4me3 + nucleosomes:DNA methylation + nucleosomes:SNPs/kb + H3K4me3:DNA methylation + H3K4me3:width + DNA methylation:width + SNPs/kb:width

The output of the model analysis is shown in the table reporting the coefficients estimate, the estimated standard error, the z-value and a two-tailed *P*-value (Pr(>|z|)) corresponding to the z ratio.

	Coefficients Estimate	Standard Error	z-value	Pr(>|z|)
(Intercept)	-4.246	0.044	-97.063	0
SPO11-1-oligos	0.048	0.047	1.0123	0.311
Nucleosomes	-0.278	0.044	-6.357	2.06×10^{-10}
H3K4me3	-0.245	0.033	-7.376	1.63×10^{-13}
DNA methylation	-1.47	0.309	-4.767	1.87×10^{-6}
SNPs/kb	-92.475	4.783	-19.334	2.74×10^{-83}
Width	1.79×10^{-4}	1.81×10^{-4}	9.862	6.06×10^{-23}
SPO11-1:Nucleosomes	0.070	0.037	1.880	0.060
SPO11-1:DNA methylation	0.506	0.261	1.942	0.052
SPO11-1:Width	1.93×10^{-4}	4.36×10^{-4}	4.419	9.93×10^{-6}
Nucleosomes: H3K4me3	-0.090	0.026	-3.447	5.67×10^{-4}
Nucleosomes:DNA methylation	-0.413	0.207	-1.989	0.047
Nucleosomes:SNPs/kb	13.106	4.386	2.988	2.80×10^{-3}
H3K4me3:DNA methylation	1.4163	0.196	7.209	5.65×10^{-13}
H3K4me3:Width	5.54×10^{-4}	2.16×10^{-4}	2.562	0.010
DNA methylation:Width	-4.06×10^{-4}	7.29×10^{-4}	-5.572	2.52×10^{-8}
SNPs/kb:Width	0.203	7.53×10^{-3}	27.003	1.38×10^{-160}

Appendix Table S6. Crossover numbers in centromeres, pericentromeres or chromosome arms in wild type and *msh2* populations. The centromeres were previously defined as crossover suppressed regions in a wild type Col×Ler F_2 population, the pericentromeres as the contiguous regions surrounding the centromeres with higher than average DNA methylation, and the remainder of the chromosomes as the arms. We used 2×2 contingency tables and X^2 tests to assess whether the proportion of crossovers in the pericentromeres and centromeres versus the arms were different between the genotypes. We observed that *msh2* crossovers in Col×Ler, Col×CLC and Col×Ct backgrounds showed significant differences to wild type (P=1.69×10^{-7}, P=1.00×10^{-11}, P=2.76×10^{-15}).

Genotype	Cross	Arm	Pericentromeres	Total
Wild type	Col×Ler	1,285	454	1,739
msh2	Col×Ler	1,166	260	1,426
Wild type	Col×CLC	971	563	1,534
msh2	Col×CLC	1,208	412	1,620
Wild type	Col×Ct	1,861	617	2,478
msh2	Col×Ct	2,269	433	2,702

Appendix Table S7. *I1b* fluorescent pollen count data for the wild type and *msh2* mutant in inbred and hybrid backgrounds. Genetic distance in centimorgans (cM) is calculated as cM = $100\times(N_Y/(N_Y+N_{R+Y}))$, where N_Y is the number of yellow alone pollen, and N_{Y+R} is the number of red and yellow pollen. X^2 tests comparing the number of recombinant (yellow alone) and non-recombinant (red and yellow) pollen counts were performed to test for significant differences between genotypes. The *I1b* FTL T-DNAs are 3,905,442-eYFP and 5,755,618-dsRed2 that define a 1.85 Mb interval located interstitially on chromosome 1.

Cross	Genotype	Total	Red alone	Red + Yellow	Non-colour	Yellow alone	cM
Col×Col	Wild type	24,624	1,104	10,015	12,651	854	7.86
Col×Col	Wild type	21,161	938	8,034	11,416	773	8.78
Col×Col	Wild type	24,760	1,052	10,053	12,742	913	8.33
Col×Col	Wild type	21,222	901	8,020	11,565	736	8.41
Col×Col	Wild type	23,913	1,087	10,105	11,771	950	8.59
Col×Col	*msh2-1*	26,220	1,187	10,021	14,036	976	8.88
Col×Col	*msh2-1*	20,792	908	8,019	11,094	771	8.77
Col×Col	*msh2-1*	8,610	365	3,188	4,746	311	8.89
Col×Ler	Wild type	17,212	719	4,455	8,595	235	5.01
Col×Ler	Wild type	13,382	473	4,064	6,346	254	5.88
Col×Ler	Wild type	24,994	960	8,147	13,308	441	5.14
Col×Ler	Wild type	24,563	990	7,969	13,422	481	5.69
Col×Ler	Wild type	23,809	927	8,144	12,262	449	5.23
Col×Ler	Wild type	25,445	909	8,008	13,107	409	4.86
Col×Ler	Wild type	23,774	1,518	6,345	13,186	390	5.79
Col×Ler	Wild type	23,064	863	8,141	11,788	422	4.93
Col×Ler	Wild type	22,946	951	8,074	12,150	462	5.41
Col×Ler	Wild type	21,778	1,978	4,844	11,616	332	6.41
Col×Ler	*msh2-1*	25,505	951	7,419	12,825	504	6.36
Col×Ler	*msh2-1*	21,105	1,271	5,877	11,013	440	6.97
Col×Ler	*msh2-1*	23,970	932	6,957	11,576	519	6.94
Col×Ler	*msh2-1*	29,605	1,138	8,073	14,903	601	6.93
Col×Ler	*msh2-1*	28,047	1,287	8,186	14,304	612	6.96
Col×Ler	*msh2-1*	24,880	1,220	7,980	13,162	594	6.93
Col×Ler	*msh2-1*	20,604	931	5,701	10,550	475	7.69
Col×Ler	*msh2-1*	28,238	1,140	7,932	13,949	575	6.76
Col×Ler	*msh2-1*	24,971	995	8,056	12,715	529	6.16
Col×CLC	Wild type	22,353	1,225	7,975	12,429	724	8.32
Col×CLC	Wild type	25,381	1,426	8,156	15,041	758	8.5
Col×CLC	Wild type	16,356	1,153	8,480	5,711	1,012	10.66
Col×CLC	Wild type	5,183	373	1,489	3,164	157	9.54
Col×CLC	Wild type	22,224	1,233	8,014	12,318	659	7.6
Col×CLC	Wild type	16,659	1,434	8,226	6,032	967	10.52
Col×CLC	Wild type	15,069	1,181	8,022	5,174	692	7.94
Col×CLC	Wild type	14,673	902	3,251	10,196	324	9.06
Col×CLC	*msh2-1*	19,658	1,740	9,000	7,020	1,898	17.42

Col×CLC	*msh2-1*	18,586	1,639	8,117	7,079	1,751	17.74
Col×CLC	*msh2-1*	18,643	1,618	8,073	7,305	1,647	16.94
Col×CLC	*msh2-1*	12,776	935	5,029	5,470	1,342	21.06
Col×CLC	*msh2-1*	21,749	1,775	8,153	9,993	1,828	18.31
Col×CLC	*msh2-1*	19,072	1,567	8,046	7,617	1,842	18.63

Appendix Table S8. *5.10* fluorescent seed count data for the wild type and *msh2* mutant in inbred and hybrid backgrounds. Genetic distance in centimorgans (cM) is calculated as cM = $100\times(1-(1-2(N_G+N_R)/N_T)^{1/2})$, where N_G is the number of green alone seeds, N_R is the number of red alone seeds and N_T is the total number of seeds of all classes analysed. X^2 tests comparing the number of recombinant and non-recombinant seed counts were performed to test for significant differences between genotypes. The *5.10* FTL T-DNAs are 6,501,045-dsRed and 13,229,304-eGFP that define a 6.73 Mb interval that spans the centromere on chromosome 3.

Cross	Genotype	Green alone	Red alone	Red and Green	Non-colour	Total seed	cM
Col×Col	Wild type	195	181	1392	342	2110	19.78
Col×Col	Wild type	106	81	663	164	1014	20.55
Col×Col	Wild type	155	119	1052	281	1607	18.82
Col×Col	Wild type	166	186	1209	248	1809	21.84
Col×Col	Wild type	165	147	1178	293	1783	19.38
Col×Col	Wild type	169	177	1359	321	2026	18.86
Col×Col	*msh2-1*	195	185	1319	319	2018	21.04
Col×Col	*msh2-1*	95	99	664	201	1059	20.4
Col×Col	*msh2-1*	123	128	982	218	1451	19.13
Col×Col	*msh2-1*	156	144	1207	327	1834	17.97
Col×Col	*msh2-1*	156	135	1070	246	1607	20.14
Col×Col	*msh2-1*	176	186	1373	390	2125	18.8
Col×Col	*msh2-1*	180	176	1425	349	2130	18.41
Col×Col	*msh2-1*	175	175	1362	347	2059	18.76
Col×Col	*msh2-1*	168	171	1191	324	1854	20.36
Col×Col	*msh2-1*	186	192	1401	339	2118	19.81
Col×Ler	Wild type	187	196	1413	361	2157	19.7
Col×Ler	Wild type	155	155	1342	297	1949	17.42
Col×Ler	Wild type	190	180	1322	291	1983	20.83
Col×Ler	Wild type	180	183	1364	336	2063	19.5
Col×Ler	Wild type	148	158	1146	291	1743	19.45
Col×Ler	Wild type	211	222	1482	327	2242	21.66
Col×Ler	Wild type	173	202	1424	368	2167	19.14
Col×Ler	Wild type	190	194	1417	339	2140	19.93
Col×Ler	Wild type	179	185	1413	307	2084	19.34
Col×Ler	Wild type	176	186	1377	385	2124	18.81
Col×Ler	*msh2-1*	147	158	1207	292	1804	18.65
Col×Ler	*msh2-1*	159	175	1441	344	2119	17.25
Col×Ler	*msh2-1*	162	168	1297	318	1945	18.72
Col×Ler	*msh2-1*	156	154	1345	343	1998	16.95
Col×Ler	*msh2-1*	160	171	1383	372	2086	17.38
Col×Ler	*msh2-1*	172	194	1315	322	2003	20.34
Col×Ler	*msh2-1*	146	109	1215	271	1741	15.91
Col×CLC	Wild type	231	219	1341	253	2044	25.19
Col×CLC	Wild type	97	95	532	122	846	26.1
Col×CLC	Wild type	199	195	1251	251	1896	23.55

Col×CLC	Wild type	204	220	1261	278	1963	24.63
Col×CLC	Wild type	191	191	1149	237	1768	24.64
Col×CLC	Wild type	233	225	1273	282	2013	26.18
Col×CLC	Wild type	252	251	1361	297	2161	26.89
Col×CLC	Wild type	217	236	1379	305	2137	24.1
Col×CLC	Wild type	218	229	1364	292	2103	24.18
Col×CLC	Wild type	209	236	1350	299	2094	24.17
Col×CLC	Wild type	236	228	1297	255	2016	26.54
Col×CLC	Wild type	231	245	1245	275	1996	27.68
Col×CLC	Wild type	248	241	1385	291	2165	25.95
Col×CLC	Wild type	218	196	1164	266	1844	25.77
Col×CLC	Wild type	230	209	1206	258	1903	26.61
Col×CLC	Wild type	170	154	1016	209	1549	23.73
Col×CLC	Wild type	211	284	1373	282	2150	26.55
Col×CLC	*msh2-1*	216	212	1283	268	1979	24.67
Col×CLC	*msh2-1*	236	236	1350	277	2099	25.82
Col×CLC	*msh2-1*	147	152	992	220	1511	22.27
Col×CLC	*msh2-1*	233	242	1387	288	2150	25.29
Col×CLC	*msh2-1*	244	240	1406	287	2177	25.48
Col×CLC	*msh2-1*	215	222	1298	298	2033	24.5
Col×CLC	*msh2-1*	245	231	1451	338	2265	23.86
Col×CLC	*msh2-1*	202	184	1182	297	1865	23.45
Col×CLC	*msh2-1*	192	211	1282	278	1963	23.23

Appendix Table S9. *CEN3* crossover frequency in wild type and *msh2* Col/Ct hybrids. Genetic distance in centimorgans (cM) is calculated as cM = $100\times(N_Y/(N_Y+N_{R+Y}))$, where N_Y is the number of yellow alone pollen, and N_{Y+R} is the number of red and yellow pollen. The *CEN3* FTL T-DNAs are 11,115,724-eYFP and 16,520,560-dsRed2 that define a 5.40 Mb interval located across the centromere of chromosome 3.

Cross	Genotype	Total	Red alone	Red + Green	Non-colour	Green alone	cM
Col x Ct	Wild type	3656	228	1237	2065	126	10.19
Col x Ct	Wild type	3175	195	1095	1790	95	8.68
Col x Ct	Wild type	13563	697	5800	6522	544	9.38
Col x Ct	Wild type	13698	812	6598	5638	650	9.85
Col x Ct	Wild type	12981	673	5840	5840	628	10.75
Col x Ct	Wild type	2133	82	752	1211	88	11.70
Col x Ct	Wild type	5593	282	2326	2805	180	7.74
Col x Ct	Wild type	4044	213	1302	2426	103	7.91
Col x Ct	Wild type	5573	260	1591	3534	188	11.82
Col x Ct	Wild type	6126	596	2251	3024	255	11.33
Col x Ct	*msh2*	14462	340	7080	6786	256	3.62
Col x Ct	*msh2*	12150	290	5170	6480	210	4.06
Col x Ct	*msh2*	3258	63	1443	1691	61	4.23
Col x Ct	*msh2*	3984	105	1884	1879	116	6.16
Col x Ct	*msh2*	3748	82	2030	1553	83	4.09
Col x Ct	*msh2*	10291	235	6054	3762	240	3.96
Col x Ct	*msh2*	6209	137	3148	2772	152	4.83
Col x Ct	*msh2*	13477	296	8408	4454	319	3.79

Appendix Table S10. Proportion of rod and ring bivalents at metaphase I in DAPI spreads from wild type and *msh2* hybrids. The five bivalents of metaphase I DAPI spreads were scored as either rod (1 chiasmata) or ring (>1 chiasma) morphology. Pairwise Wilcoxon rank sum tests were performed, using the Benjamini & Hochberg correction for multiple testing, to test for significant differences between genotypes. Wild type and *msh2* were not significantly different in Col×Ler (*P*=0.957) or Col×CLC (*P*=0.234) backgrounds.

Individual	Rod	Ring	Proportion rod	Proportion ring
Col×Ler wild type	3	2	0.6	0.4
Col×Ler wild type	2	3	0.4	0.6
Col×Ler wild type	2	3	0.4	0.6
Col×Ler wild type	2	3	0.4	0.6
Col×Ler wild type	4	1	0.8	0.2
Col×Ler wild type	2	3	0.4	0.6
Col×Ler wild type	4	1	0.8	0.2
Col×Ler wild type	2	3	0.4	0.6
Col×Ler wild type	2	3	0.4	0.6
Col×Ler wild type	3	2	0.6	0.4
Col×Ler wild type	2	3	0.4	0.6
Col×Ler wild type	2	3	0.4	0.6
Col×Ler wild type	4	1	0.8	0.2
Col×Ler wild type	2	3	0.4	0.6
Col×Ler wild type	3	2	0.6	0.4
Col×Ler wild type	3	2	0.6	0.4
Col×Ler wild type	3	2	0.6	0.4
Col×Ler wild type	2	3	0.4	0.6
Col×Ler wild type	5	0	1	0
Col×Ler wild type	4	1	0.8	0.2
Total	56	44	0.56	0.44
Col×Ler *msh2-1*	3	2	0.6	0.4
Col×Ler *msh2-1*	3	2	0.6	0.4
Col×Ler *msh2-1*	2	3	0.4	0.6
Col×Ler *msh2-1*	4	1	0.8	0.2
Col×Ler *msh2-1*	4	1	0.8	0.2
Col×Ler *msh2-1*	1	4	0.2	0.8
Col×Ler *msh2-1*	3	2	0.6	0.4
Col×Ler *msh2-1*	2	3	0.4	0.6
Total	22	18	0.55	0.45
Col×CLC wild type	1	4	0.2	0.8
Col×CLC wild type	1	4	0.2	0.8
Col×CLC wild type	0	5	0	1
Col×CLC wild type	2	3	0.4	0.6
Col×CLC wild type	0	5	0	1
Col×CLC wild type	0	5	0	1
Col×CLC wild type	2	3	0.4	0.6
Total	6	29	0.17	0.83
Col×CLC *msh2-1*	1	4	0.2	0.8

Col×CLC *msh2-1*	2	3	0.4	0.6
Col×CLC *msh2-1*	2	3	0.4	0.6
Col×CLC *msh2-1*	1	4	0.2	0.8
Col×CLC *msh2-1*	1	4	0.2	0.8
Col×CLC *msh2-1*	2	3	0.4	0.6
Total	9	21	0.3	0.7

Appendix Table S11. Alexander staining pollen viability counts in wild type and *msh2* mutants in inbred and hybrid backgrounds. Pollen viability assays were performed on an average of nine plants per genotype. Viable pollen grains stain purple, whereas inviable pollen is misshapen and stains blue-green. 2×2 contingency tables were constructed using aggregate viable and inviable pollen, and X^2 tests were performed to test for statistically significant differences between wild type and *msh2*.

Individual	Viable	Inviable	Total	% viable	% inviable	*P*-value
Col×Col wild type	1,498	24	1,522	98.4	1.6	
Col×Col wild type	1,484	26	1,510	98.3	1.7	
Col×Col wild type	1,712	16	1,728	99.1	0.9	
Col×Col wild type	1,591	14	1,605	99.1	0.9	
Col×Col wild type	1,837	20	1,857	98.9	1.1	
Col×Col wild type	1,793	15	1,808	99.2	0.8	
Col×Col wild type	1,972	27	1,999	98.6	1.4	
Total	11,887	142	12,029	98.8	1.2	-
Col×Col *msh2-1*	1,464	36	1,500	97.6	2.4	
Col×Col *msh2-1*	1,398	40	1,438	97.2	2.8	
Col×Col *msh2-1*	1,361	26	1,387	98.1	1.9	
Col×Col *msh2-1*	1,683	47	1,730	97.3	2.7	
Col×Col *msh2-1*	1,831	62	1,893	96.7	3.3	
Col×Col *msh2-1*	1,892	39	1,931	98.0	2.0	
Col×Col *msh2-1*	1,995	23	2,018	98.9	1.1	
Total	11,624	273	11,897	97.7	2.3	5.7×10^{-11}
Ler×Ler wild type	1,420	55	1,475	96.3	3.7	
Ler×Ler wild type	2,000	4	2,004	99.8	0.2	
Ler×Ler wild type	2,089	5	2,094	99.8	0.2	
Ler×Ler wild type	2,147	11	2,158	99.5	0.5	
Ler×Ler wild type	2,016	22	2,038	98.9	1.1	
Ler×Ler wild type	2,016	6	2,022	99.7	0.3	
Ler×Ler wild type	2,015	11	2,026	99.5	0.5	
Ler×Ler wild type	2,135	8	2,143	99.6	0.4	
Ler×Ler wild type	2,038	7	2,045	99.7	0.3	
Ler×Ler wild type	2,012	14	2,026	99.3	0.7	
Ler×Ler wild type	2,009	7	2,016	99.7	0.3	
Total	21,897	150	22,047	99.2	0.8	-
Ler×Ler *msh2-1*	2,309	65	2,374	97.3	2.7	
Ler×Ler *msh2-1*	2,032	50	2,082	97.6	2.4	
Ler×Ler *msh2-1*	2,043	85	2,128	96.0	4.0	
Ler×Ler *msh2-1*	921	37	958	96.1	3.9	
Ler×Ler *msh2-1*	2,032	61	2,093	97.1	2.9	
Ler×Ler *msh2-1*	2,004	128	2,132	94.0	6.0	
Ler×Ler *msh2-1*	2,031	41	2,072	98.0	2.0	
Ler×Ler *msh2-1*	2,061	40	2,101	98.1	1.9	
Ler×Ler *msh2-1*	1,981	179	2,160	91.7	8.3	
Total	17,414	686	18,100	96.2	3.8	$< 2.2\times10^{-16}$
CLC×CLC wild type	1,357	6	1,363	99.6	0.4	
CLC×CLC wild type	1,343	11	1,354	99.2	0.8	

CLC×CLC wild type	1,612	3	1,615	99.8	0.2	
CLC×CLC wild type	1,629	3	1,632	99.8	0.2	
CLC×CLC wild type	1,642	7	1,649	99.6	0.4	
CLC×CLC wild type	1,346	9	1,355	99.3	0.7	
CLC×CLC wild type	1,706	12	1,718	99.3	0.7	
CLC×CLC wild type	1,199	13	1,212	98.9	1.1	
CLC×CLC wild type	1,636	19	1,655	98.9	1.1	
CLC×CLC wild type	1,206	9	1,215	99.3	0.7	
CLC×CLC wild type	1,721	14	1,735	99.2	0.8	
Total	16,397	106	16,503	99.3	0.7	-
CLC×CLC *msh2-1*	1,866	29	1,895	98.5	1.5	
CLC×CLC *msh2-1*	1,841	39	1,880	97.9	2.1	
CLC×CLC *msh2-1*	1,866	9	1,875	99.5	0.5	
CLC×CLC *msh2-1*	1,917	13	1,930	99.3	0.7	
CLC×CLC *msh2-1*	1,835	85	1,920	95.6	4.4	
CLC×CLC *msh2-1*	1,406	15	1,421	98.9	1.1	
CLC×CLC *msh2-1*	1,938	14	1,952	99.3	0.7	
CLC×CLC *msh2-1*	1,739	221	1,960	88.7	11.3	
CLC×CLC *msh2-1*	1,906	114	2,020	94.4	5.6	
Total	16,314	539	16,853	96.9	3.1	$< 2.2\times10^{-16}$
Col×CLC wild type	2,054	40	2,094	98.1	1.9	
Col×CLC wild type	1,857	18	1,875	99.0	1.0	
Col×CLC wild type	1,950	47	1,997	97.6	2.4	
Col×CLC wild type	2,025	35	2,060	98.3	1.7	
Col×CLC wild type	2,000	51	2,051	97.5	2.5	
Col×CLC wild type	2,046	56	2,102	97.3	2.7	
Col×CLC wild type	1,995	23	2,018	98.9	1.1	
Col×CLC wild type	1,462	23	1,485	98.5	1.5	
Total	15,389	293	15,682	98.2	1.8	-
Col×CLC *msh2-1*	2,037	44	2,081	97.9	2.1	
Col×CLC *msh2-1*	1,836	48	1,884	97.5	2.5	
Col×CLC *msh2-1*	1,856	70	1,926	96.4	3.6	
Col×CLC *msh2-1*	1,917	34	1,951	98.3	1.7	
Col×CLC *msh2-1*	2,076	56	2,132	97.4	2.6	
Col×CLC *msh2-1*	2,088	50	2,138	97.7	2.3	
Col×CLC *msh2-1*	1,997	49	2,046	97.6	2.4	
Col×CLC *msh2-1*	2,212	73	2,285	96.8	3.2	
Col×CLC *msh2-1*	2,032	117	2,149	94.6	5.4	
Col×CLC *msh2-1*	2,031	57	2,088	97.3	2.7	
Col×CLC *msh2-1*	2,178	42	2,220	98.1	1.9	
Col×CLC *msh2-1*	1,968	28	1,996	98.6	1.4	
Total	24,228	668	24,896	97.3	2.7	5.18×10^{-8}

Appendix Table S12. *420* fluorescent seed count data for the wild type and *msh2* mutants in recombinant lines with differing pattern of heterozygosity. The following recombinant backgrounds were used: 'HOM-HOM' that are Col/Col inbred throughout the genome, 'HET-HET' that are Col/Ct heterozygous throughout the genome, 'HET-HOM' where the *420* region is Col/Ct heterozygous and the remainder of chromosome 3 is Col/Col homozygous, and 'HOM-HET' where *420* is Col/Col homozygous and the remainder of chromosome 3 is Col/Ct heterozygous**.** Genetic distance in centimorgans (cM) is calculated as $cM = 100\times(1-(1-2(N_G+N_R)/N_T)^{1/2})$, where N_G is the number of green alone seeds, N_R is the number of red alone seeds and N_T is the total number of seeds of all classes analysed. t-tests comparing the *420* recombination frequency between the groups were performed to test for significant differences between genotypes. The *420* FTL T-DNAs are 256,516-eGFP and 5,361,637-dsRed that define a 5.11 Mb interval that spans a subtelomeric region on chromosome 3.

Cross	Genotype	Green alone	Red alone	Red and Green	Non-colour	Total seed	cM
HOM-HOM	Wild type	164	176	1301	356	1997	18.79
HOM-HOM	Wild type	122	108	968	271	1469	17.12
HOM-HOM	Wild type	165	155	1344	367	2031	17.24
HOM-HOM	Wild type	186	153	1448	380	2167	17.11
HOM-HOM	Wild type	224	199	1470	394	2287	20.62
HOM-HOM	Wild type	188	177	1451	410	2226	18.02
HOM-HOM	Wild type	152	151	1332	377	2012	16.41
HOM-HOM	Wild type	150	126	1147	320	1743	17.34
HOM-HOM	Wild type	131	145	1202	324	1802	16.71
HOM-HOM	Wild type	171	149	1269	340	1929	18.26
HOM-HOM	Wild type	149	112	1058	274	1593	18.01
HOM-HOM	Wild type	93	119	837	193	1242	18.84
HOM-HOM	Wild type	139	119	1030	257	1545	18.39
HOM-HOM	Wild type	155	130	1064	245	1594	19.85
HOM-HOM	Wild type	182	203	1676	399	2460	17.12
HOM-HOM	Wild type	147	176	1447	350	2120	16.62
HOM-HOM	Wild type	129	131	1150	260	1670	17.02
HOM-HOM	Wild type	180	205	1571	427	2383	17.73
HOM-HOM	Wild type	146	175	1354	323	1998	17.62
HOM-HOM	Wild type	108	114	850	198	1270	19.35
HOM-HOM	Wild type	180	224	1418	334	2156	20.93
HOM-HOM	Wild type	162	168	1353	327	2010	18.05
HOM-HOM	Wild type	204	196	1462	398	2260	19.62
HOM-HOM	Wild type	158	172	1440	346	2116	17.05
HOM-HOM	Wild type	214	228	1486	366	2294	21.60
HOM-HOM	*msh2-2*	65	48	477	59	649	19.27
HOM-HOM	*msh2-2*	188	172	1378	400	2138	18.56
HOM-HOM	*msh2-2*	220	177	1303	355	2055	21.67
HOM-HOM	*msh2-2*	166	109	929	238	1442	21.35
HOM-HOM	*msh2-2*	209	185	1345	362	2101	20.95
HOM-HOM	*msh2-2*	105	146	984	235	1470	18.85
HOM-HOM	*msh2-2*	191	133	1273	357	1954	18.25
HOM-HOM	*msh2-2*	98	94	730	188	1110	19.13
HOM-HOM	*msh2-2*	34	38	235	51	358	22.68
HOM-HOM	*msh2-2*	102	100	796	189	1187	18.78
HOM-HOM	*msh2-2*	162	186	1288	310	1946	19.85
HOM-HOM	*msh2-2*	165	166	1258	357	1946	18.77
HOM-HOM	*msh2-2*	152	137	1042	287	1618	19.83
HOM-HOM	*msh2-2*	164	170	1155	286	1775	21.03
HOM-HOM	*msh2-2*	165	177	1460	340	2142	17.50
HOM-HOM	*msh2-2*	194	200	1322	298	2014	21.98
HOM-HOM	*msh2-2*	155	142	1198	323	1818	17.95
HOM-HOM	*msh2-2*	149	172	1190	297	1808	19.69

HOM-HOM	*msh2-2*	191	208	1547	359	2305	19.14
HET-HET	Wild type	146	135	1390	385	2056	14.76
HET-HET	Wild type	155	164	1400	408	2127	16.33
HET-HET	Wild type	147	149	1406	414	2116	15.13
HET-HET	Wild type	115	159	1248	325	1847	16.14
HET-HET	Wild type	151	135	1225	339	1850	16.88
HET-HET	Wild type	113	134	1126	301	1674	16.04
HET-HET	Wild type	140	145	1454	357	2096	14.67
HET-HET	Wild type	133	137	1296	388	1954	14.93
HET-HET	Wild type	161	136	1445	398	2140	15.00
HET-HET	Wild type	158	162	1589	478	2387	14.45
HET-HET	Wild type	167	184	1463	418	2232	17.21
HET-HET	Wild type	138	106	1263	357	1864	14.08
HET-HET	Wild type	101	108	920	211	1340	17.05
HET-HET	Wild type	140	156	1337	391	2024	15.89
HET-HET	Wild type	145	154	1260	325	1884	17.38
HET-HET	Wild type	179	143	1450	402	2174	16.11
HET-HET	*msh2-3*	89	86	648	145	968	20.10
HET-HET	*msh2-3*	137	116	888	223	1364	20.69
HET-HET	*msh2-3*	140	136	960	211	1447	21.35
HET-HET	*msh2-3*	68	72	532	137	809	19.14
HET-HET	*msh2-3*	112	103	696	186	1097	22.02
HET-HET	*msh2-3*	105	100	877	209	1291	17.39
HET-HET	*msh2-3*	89	109	854	213	1265	17.12
HET-HET	*msh2-3*	130	175	1080	261	1646	20.66
HET-HET	*msh2-3*	136	134	1123	253	1646	18.03
HET-HET	*msh2-3*	143	149	1195	274	1761	18.25
HET-HET	*msh2-3*	133	113	981	240	1467	18.48
HET-HET	*msh2-3*	154	157	1065	282	1658	20.95
HET-HET	*msh2-3*	86	86	610	129	911	21.11
HET-HET	*msh2-3*	70	65	410	95	640	23.97
HET-HET	*msh2-3*	107	100	660	174	1041	22.39
HET-HET	*msh2-3*	128	115	859	203	1305	20.78
HET-HET	*msh2-3*	86	86	610	129	911	21.11
HET-HET	*msh2-3*	85	96	628	145	954	21.23
HET-HET	*msh2-3*	91	93	590	158	932	22.21
HET-HET	*msh2-3*	125	131	916	204	1376	20.76
HET-HOM	Wild type	264	259	1511	330	2364	25.33
HET-HOM	Wild type	257	295	1381	335	2268	28.36
HET-HOM	Wild type	298	265	1432	423	2418	26.90
HET-HOM	Wild type	271	239	1355	273	2138	27.69
HET-HOM	Wild type	299	259	1495	459	2512	25.45
HET-HOM	Wild type	238	232	1222	222	1914	28.66
HET-HOM	Wild type	232	266	1402	283	2183	26.26
HET-HOM	Wild type	236	239	1378	295	2148	25.32
HET-HOM	Wild type	236	287	1332	267	2122	28.79
HET-HOM	Wild type	173	235	1268	299	1975	23.39
HET-HOM	Wild type	209	267	1379	302	2157	25.26
HET-HOM	Wild type	279	239	1483	382	2383	24.82
HET-HOM	Wild type	258	264	1427	353	2302	26.08
HET-HOM	Wild type	246	278	1440	284	2248	26.94
HET-HOM	Wild type	253	242	1487	339	2321	24.27
HET-HOM	*msh2-4*	184	150	1316	354	2004	18.35
HET-HOM	*msh2-4*	198	194	1475	356	2223	19.54
HET-HOM	*msh2-4*	154	194	1608	383	2339	16.19
HET-HOM	*msh2-4*	200	180	1489	412	2281	18.34
HET-HOM	*msh2-4*	163	158	1413	319	2053	17.10
HET-HOM	*msh2-4*	177	176	1506	386	2245	17.20
HET-HOM	*msh2-4*	173	181	1447	386	2187	17.76
HET-HOM	*msh2-4*	197	159	1326	371	2053	19.18

HET-HOM	*msh2-4*	200	160	1366	314	2040	19.56
HET-HOM	*msh2-4*	164	185	1513	387	2249	16.96
HET-HOM	*msh2-4*	162	166	1141	302	1771	20.65
HET-HOM	*msh2-4*	195	195	1549	354	2293	18.77
HET-HOM	*msh2-4*	175	216	1545	361	2297	18.79
HET-HOM	*msh2-4*	172	166	1414	344	2096	17.69
HET-HOM	*msh2-4*	199	174	1578	414	2365	17.26
HET-HOM	*msh2-4*	181	208	1534	420	2343	18.27
HET-HOM	*msh2-4*	160	190	1399	333	2082	18.53
HET-HOM	*msh2-4*	179	152	1362	329	2022	17.99
HET-HOM	*msh2-4*	174	187	1297	355	2013	19.92
HOM-HET	Wild type	101	104	1417	427	2049	10.56
HOM-HET	Wild type	97	114	1419	397	2027	11.02
HOM-HET	Wild type	123	81	1269	369	1842	11.77
HOM-HET	Wild type	92	100	1318	398	1908	10.63
HOM-HET	Wild type	82	123	1437	427	2069	10.45
HOM-HET	Wild type	65	57	970	273	1365	9.38
HOM-HET	Wild type	99	107	1529	451	2186	9.92
HOM-HET	Wild type	83	114	1423	431	2051	10.12
HOM-HET	Wild type	105	100	1321	409	1935	11.22
HOM-HET	Wild type	69	62	1149	325	1605	8.53
HOM-HET	Wild type	64	89	999	284	1436	11.29
HOM-HET	Wild type	100	123	1511	448	2182	10.80
HOM-HET	Wild type	52	55	738	197	1042	10.86
HOM-HET	*msh2-5*	164	148	897	202	1411	25.32
HOM-HET	*msh2-5*	218	233	1386	356	2193	23.27
HOM-HET	*msh2-5*	142	156	946	210	1454	23.18
HOM-HET	*msh2-5*	213	196	1350	315	2074	22.18
HOM-HET	*msh2-5*	170	170	1121	240	1701	22.53
HOM-HET	*msh2-5*	220	234	1223	235	1912	27.54
HOM-HET	*msh2-5*	191	169	1063	250	1673	24.53
HOM-HET	*msh2-5*	200	246	1477	356	2279	21.99
HOM-HET	*msh2-5*	175	173	1085	244	1677	23.52
HOM-HET	*msh2-5*	155	178	1083	235	1651	22.76
HOM-HET	*msh2-5*	248	227	1432	303	2210	24.49
HOM-HET	*msh2-5*	171	207	1074	252	1704	25.41
HOM-HET	*msh2-5*	192	192	1213	263	1860	23.38
HOM-HET	*msh2-5*	209	192	1171	303	1875	24.35
HOM-HET	*msh2-5*	120	98	699	175	1092	22.49
HOM-HET	*msh2-5*	94	84	604	165	947	21.00
HOM-HET	*msh2-5*	149	167	1222	347	1885	18.47
HOM-HET	*msh2-5*	165	195	1334	357	2051	19.44
HOM-HET	*msh2-5*	127	142	857	201	1327	22.89
HOM-HET	*msh2-5*	142	130	897	236	1405	21.72
HOM-HET	*msh2-5*	96	115	678	175	1064	22.32
HOM-HET	*msh2-5*	86	106	719	164	1075	19.83
HOM-HET	*msh2-5*	224	201	1323	322	2070	23.23
HOM-HET	*msh2-5*	151	201	1272	309	1933	20.26
HOM-HET	*msh2-5*	135	198	1203	307	1843	20.09
HOM-HET	*msh2-5*	118	105	757	204	1184	21.05

Appendix Table S13. ***420*** **fluorescent seed count data for wild type and** ***HEI10-OE*** **in recombinant lines differing in the pattern of heterozygosity.** The following recombinant backgrounds were used: 'HOM-HOM' that are Col/Col inbred throughout the genome, 'HET-HET' that are Col/Ct heterozygous throughout the genome, 'HET-HOM' where the *420* region is Col/Ct heterozygous and the remainder of chromosome 3 is Col/Col homozygous, and 'HOM-HET' where *420* is Col/Col homozygous and the remainder of chromosome 3 is Col/Ct heterozygous**.** Genetic distance in centimorgans (cM) is calculated as cM = $100\times(1-(1-2(N_G+N_R)/N_T)^{1/2})$, where N_G is the number of green alone seeds, N_R is the number of red alone seeds and N_T is the total number of seeds of all classes analysed. T-test comparing the *420* recombination frequency between the groups were performed to test for significant differences between genotypes. The *420* FTL T-DNAs are 256,516-eGFP and 5,361,637-dsRed that define a 5.11 Mb interval that spans a subtelomeric region on chromosome 3.

Cross	Genotype	Green alone	Red alone	Red and Green	Non-colour	Total seed	cM
HOM-HOM	Wild type	443	411	2730	676	4260	22.60
HOM-HOM	Wild type	313	335	2633	710	3991	17.83
HOM-HOM	Wild type	355	352	2486	631	3824	20.61
HOM-HOM	Wild type	330	361	2374	681	3746	20.56
HOM-HOM	Wild type	351	343	2268	530	3492	22.38
HOM-HOM	Wild type	338	349	2629	648	3964	19.17
HOM-HOM	Wild type	347	417	2548	671	3983	21.49
HOM-HOM	Wild type	324	276	2089	536	3225	20.76
HOM-HOM	Wild type	385	412	3069	757	4623	19.06
HOM-HOM	Wild type	73	80	579	136	868	19.53
HOM-HOM	Wild type	324	321	2305	636	3586	19.98
HOM-HOM	Wild type	381	313	2519	667	3880	19.86
HOM-HOM	Wild type	320	310	2186	636	3452	20.31
HOM-HOM	Wild type	337	374	2588	683	3982	19.82
HOM-HOM	Wild type	372	334	2526	689	3921	20.01
HOM-HOM	Wild type	350	307	2448	683	3788	19.18
HOM-HOM	Wild type	248	248	1734	454	2684	20.60
HOM-HOM	Wild type	292	290	2310	596	3488	18.37
HOM-HOM	Wild type	376	324	2450	563	3713	21.07
HOM-HOM	Wild type	311	262	2175	590	3338	18.96
HOM-HOM	Wild type	128	128	969	222	1447	19.62
HOM-HOM	*HEI10-OE*	296	323	1178	211	2008	38.08
HOM-HOM	*HEI10-OE*	307	333	1253	220	2113	37.21
HOM-HOM	*HEI10-OE*	307	355	1270	201	2133	38.41
HOM-HOM	*HEI10-OE*	324	328	1276	217	2145	37.38
HOM-HOM	*HEI10-OE*	285	290	1205	214	1994	34.94
HOM-HOM	*HEI10-OE*	279	274	1250	273	2076	31.64
HOM-HOM	*HEI10-OE*	262	297	1204	196	1959	34.48
HOM-HOM	*HEI10-OE*	300	319	1236	183	2038	37.35
HOM-HOM	*HEI10-OE*	346	328	1316	200	2190	37.99
HOM-HOM	*HEI10-OE*	319	346	1234	192	2091	39.67
HOM-HOM	*HEI10-OE*	471	551	2091	411	3524	35.19
HOM-HOM	*HEI10-OE*	551	684	2492	443	4170	36.15
HOM-HOM	*HEI10-OE*	528	617	2198	418	3761	37.46
HOM-HOM	*HEI10-OE*	365	415	1589	242	2611	36.55
HOM-HOM	*HEI10-OE*	300	250	1273	206	2029	32.33
HOM-HOM	*HEI10-OE*	488	558	2054	401	3501	36.56
HOM-HOM	*HEI10-OE*	595	596	2307	398	3896	37.66
HOM-HOM	*HEI10-OE*	273	234	956	173	1636	38.34
HET-HET	Wild type	373	328	2825	734	4260	18.09
HET-HET	Wild type	273	266	2335	645	3519	16.71
HET-HET	Wild type	318	324	2736	721	4099	17.13
HET-HET	Wild type	400	326	2833	743	4302	18.61
HET-HET	Wild type	257	239	2194	656	3346	16.12
HET-HET	Wild type	191	155	1355	355	2056	18.55
HET-HET	Wild type	345	318	2533	733	3929	18.61
HET-HET	Wild type	277	327	2345	607	3556	18.74
HET-HET	Wild type	295	321	2433	669	3718	18.23
HET-HET	Wild type	142	158	1234	335	1869	17.60
HET-HET	Wild type	293	316	2272	509	3390	19.96
HET-HET	Wild type	210	237	1810	461	2718	18.08

HET-HET	Wild type	303	308	2454	679	3744	17.93
HET-HET	Wild type	306	310	2258	640	3514	19.41
HET-HET	Wild type	338	374	2879	823	4414	17.70
HET-HET	Wild type	392	357	2602	696	4047	20.64
HET-HET	Wild type	405	422	2763	687	4277	21.69
HET-HET	Wild type	364	378	2270	580	3592	23.39
HET-HET	*HEI10-OE*	624	549	2283	386	3842	37.60
HET-HET	*HEI10-OE*	646	562	2185	389	3782	39.90
HET-HET	*HEI10-OE*	540	626	2223	438	3827	37.50
HET-HET	*HEI10-OE*	438	377	1446	257	2518	40.61
HET-HET	*HEI10-OE*	336	358	1432	226	2352	35.98
HET-HET	*HEI10-OE*	233	252	902	150	1537	39.26
HET-HET	*HEI10-OE*	448	541	1902	349	3240	37.59
HET-HET	*HEI10-OE*	513	453	1844	298	3108	38.49
HET-HET	*HEI10-OE*	603	596	2159	435	3793	39.35
HET-HOM	Wild type	195	181	1150	255	1781	23.99
HET-HOM	Wild type	177	164	1065	229	1635	23.65
HET-HOM	Wild type	193	176	1119	257	1745	24.03
HET-HOM	Wild type	102	113	634	135	984	24.97
HET-HOM	Wild type	63	66	422	98	649	22.38
HET-HOM	Wild type	196	247	1269	258	1970	25.82
HET-HOM	Wild type	225	221	1315	296	2057	24.74
HET-HOM	Wild type	124	103	602	120	949	27.78
HET-HOM	Wild type	475	422	2396	603	3896	26.55
HET-HOM	Wild type	446	392	2628	607	4073	23.29
HET-HOM	Wild type	458	410	2332	541	3741	26.79
HET-HOM	Wild type	416	410	2380	635	3841	24.51
HET-HOM	Wild type	416	462	2468	585	3931	25.62
HET-HOM	Wild type	366	376	2177	457	3376	25.14
HET-HOM	Wild type	462	438	2509	588	3997	25.86
HET-HOM	Wild type	357	345	1896	439	3037	26.67
HET-HOM	Wild type	430	432	2269	521	3652	27.34
HET-HOM	Wild type	402	358	2232	540	3532	24.52
HET-HOM	Wild type	142	136	940	193	1411	22.16
HET-HOM	Wild type	351	411	2196	546	3504	24.83
HET-HOM	*HEI10-OE*	380	362	1132	173	2047	47.56
HET-HOM	*HEI10-OE*	324	336	1086	140	1886	45.22
HET-HOM	*HEI10-OE*	360	317	1099	168	1944	44.91
HET-HOM	*HEI10-OE*	300	238	823	120	1481	47.71
HET-HOM	*HEI10-OE*	263	236	815	118	1432	44.95
HET-HOM	*HEI10-OE*	348	327	1125	167	1967	43.99
HET-HOM	*HEI10-OE*	342	338	1061	142	1883	47.30
HET-HOM	*HEI10-OE*	310	304	966	164	1744	45.61
HET-HOM	*HEI10-OE*	737	662	2175	250	3824	48.20
HET-HOM	*HEI10-OE*	729	721	2263	390	4103	45.85
HET-HOM	*HEI10-OE*	218	187	697	111	1213	42.36
HET-HOM	*HEI10-OE*	129	141	443	67	780	44.53
HET-HOM	*HEI10-OE*	386	314	1083	138	1921	47.92
HET-HOM	*HEI10-OE*	302	261	972	163	1698	41.96
HET-HOM	*HEI10-OE*	169	173	586	96	1024	42.38
HET-HOM	*HEI10-OE*	669	619	2096	316	3700	44.88
HET-HOM	*HEI10-OE*	786	699	2309	363	4157	46.56
HET-HOM	*HEI10-OE*	657	685	2208	378	3928	43.72
HET-HOM	*HEI10-OE*	678	652	2137	341	3808	45.09
HET-HOM	*HEI10-OE*	744	674	2200	311	3929	47.26
HET-HOM	*HEI10-OE*	675	728	2268	401	4072	44.24
HOM-HET	Wild type	148	155	2110	688	3101	10.30
HOM-HET	Wild type	244	258	2491	743	3736	14.49
HOM-HET	Wild type	123	134	1265	330	1852	15.00
HOM-HET	Wild type	216	197	2168	607	3188	13.92
HOM-HET	Wild type	194	198	2475	747	3614	11.51
HOM-HET	Wild type	175	155	2343	768	3441	10.10
HOM-HET	Wild type	51	54	471	144	720	15.84
HOM-HET	Wild type	142	171	1737	517	2567	13.04
HOM-HET	Wild type	189	234	2345	689	3457	13.09
HOM-HET	Wild type	79	131	1152	330	1692	13.30
HOM-HET	Wild type	180	212	2561	690	3643	11.41
HOM-HET	Wild type	177	167	1762	546	2652	13.94
HOM-HET	Wild type	177	167	1762	546	2652	13.94
HOM-HET	Wild type	190	210	2411	751	3562	11.94
HOM-HET	Wild type	149	136	1901	608	2794	10.78

HOM-HET	Wild type	211	233	2651	782	3877	12.20
HOM-HET	Wild type	208	199	2235	674	3316	13.14
HOM-HET	Wild type	115	131	1357	444	2047	12.84
HOM-HET	Wild type	176	142	1755	565	2638	12.88
HOM-HET	Wild type	179	284	2998	859	4320	11.36
HOM-HET	Wild type	281	188	2673	817	3959	12.65
HOM-HET	Wild type	210	307	2838	860	4215	13.13
HOM-HET	Wild type	210	209	2850	793	4062	10.91
HOM-HET	Wild type	179	220	2762	785	3946	10.68
HOM-HET	Wild type	164	188	2497	802	3651	10.16
HOM-HET	Wild type	194	217	2585	811	3807	11.45
HOM-HET	Wild type	197	212	2436	746	3591	12.12
HOM-HET	Wild type	241	229	2909	864	4243	11.77
HOM-HET	Wild type	206	201	2425	699	3531	12.28
HOM-HET	Wild type	132	157	1656	429	2374	13.02
HOM-HET	Wild type	125	152	1673	521	2471	11.92
HOM-HET	*HEI10-OE*	232	167	917	164	1480	32.12
HOM-HET	*HEI10-OE*	287	294	1420	228	2229	30.81
HOM-HET	*HEI10-OE*	204	209	1011	197	1621	29.97
HOM-HET	*HEI10-OE*	205	215	999	239	1658	29.76
HOM-HET	*HEI10-OE*	191	198	1030	193	1612	28.07
HOM-HET	*HEI10-OE*	188	199	1008	205	1600	28.15
HOM-HET	*HEI10-OE*	108	131	659	146	1044	26.37
HOM-HET	*HEI10-OE*	108	105	638	118	969	25.14
HOM-HET	*HEI10-OE*	269	234	1270	255	2028	29.01
HOM-HET	*HEI10-OE*	219	221	1114	213	1767	29.15
HOM-HET	*HEI10-OE*	250	260	1173	199	1882	32.32
HOM-HET	*HEI10-OE*	233	247	1112	213	1805	31.58
HOM-HET	*HEI10-OE*	243	269	1314	252	2078	28.78
HOM-HET	*HEI10-OE*	120	102	552	96	870	30.02
HOM-HET	*HEI10-OE*	317	324	1355	222	2218	35.04
HOM-HET	*HEI10-OE*	272	251	1345	241	2109	29.00
HOM-HET	*HEI10-OE*	184	235	1152	229	1800	26.89
HOM-HET	*HEI10-OE*	99	116	543	102	860	29.29
HOM-HET	*HEI10-OE*	548	538	2505	502	4093	31.49
HOM-HET	*HEI10-OE*	373	415	1916	419	3123	29.62
HOM-HET	*HEI10-OE*	425	405	2078	429	3337	29.11
HOM-HET	*HEI10-OE*	246	252	1339	273	2110	27.34
HOM-HET	*HEI10-OE*	369	303	1678	345	2695	29.20
HOM-HET	*HEI10-OE*	481	562	2544	585	4172	29.29
HOM-HET	*HEI10-OE*	525	519	2275	478	3797	32.91
HOM-HET	*HEI10-OE*	521	493	2392	559	3965	30.11
HOM-HET	*HEI10-OE*	539	532	2581	477	4129	30.63
HOM-HET	*HEI10-OE*	440	586	2270	455	3751	32.70
HOM-HET	*HEI10-OE*	510	588	2579	444	4121	31.65
HOM-HET	*HEI10-OE*	491	594	2563	480	4128	31.13
HOM-HET	*HEI10-OE*	561	625	2419	478	4083	35.27
HOM-HET	*HEI10-OE*	468	561	2453	524	4006	30.27
HOM-HET	*HEI10-OE*	209	265	1163	221	1858	30.02
HOM-HET	*HEI10-OE*	481	568	2533	479	4061	30.47
HOM-HET	*HEI10-OE*	194	160	756	139	1249	34.19
HOM-HET	*HEI10-OE*	512	576	2212	428	3728	35.48
